# Patients Reported Adverse Effects of Antidepressants Among Depressive Disorder and Associated Risk Factors: A Multicenter Cross‐Sectional Study

**DOI:** 10.1155/da/9957695

**Published:** 2026-02-23

**Authors:** Gashaw Sisay Chanie, Gebremariam Wulie Geremew, Alemante Tafese Beyna, Melshew Fenta Misker, Habtamu Semagne Ayele, Abebe Worku Teshager, Girma Medfu Takele, Gizachew Kassahun Bizuneh, Jember Azanaw, Eyayaw Ashete Belachew, Wudneh Simegn

**Affiliations:** ^1^ Department of Clinical Pharmacy, School of Pharmacy, College of Medicine and Health Sciences, University of Gondar, Gondar, Ethiopia, uog.edu.et; ^2^ Department of Social and Clinical Pharmacy, Faculty of Pharmacy, Charles University, Praque, Czech Republic, cuni.cz; ^3^ Department of Pharmacology, School of Pharmacy, College of Medicine and Health Sciences, University of Gondar, Gondar, Ethiopia, uog.edu.et; ^4^ Department of Internal Medicine, School of Medicine, College of Medicine and Health Sciences, University of Gondar, Gondar, Ethiopia, uog.edu.et; ^5^ Department of Psychiatry, College of Medicine and Health Sciences, University of Gondar, Gondar, Ethiopia, uog.edu.et; ^6^ Department of Pharmacognosy, College of Medicine and Health Sciences, University of Gondar, Gondar, Ethiopia, uog.edu.et; ^7^ Department of Environmental and Occupational Health and Safety, Institute of Public Health, College of Medicine and Health Sciences, University of Gondar, Gondar, Ethiopia, uog.edu.et; ^8^ Department of Social and Administrative Pharmacy, School of Pharmacy, College of Medicine and Health Sciences, University of Gondar, Gondar, Ethiopia, uog.edu.et

**Keywords:** adverse effects, antidepressant, depressive disorder, Ethiopia, prevalence

## Abstract

**Background:**

Over the last two decades, antidepressant usage has seen a notable rise. Following the initiation of antidepressant medication, numerous patients experience adverse drug reactions. The main objective of this research was to determine the adverse effects of antidepressants, and identify the associated factors for outpatients who had been diagnosed with depression in three chosen clinics specializing in psychiatry in Ethiopia.

**Methods:**

A prospective multicenter cross‐sectional study was carried out from June 12, 2024, to November 13, 2024, involving a total of 422 participants. An antidepressant side effect checklist (ASEC) was employed to evaluate side effects, and unstructured questionnaires were reviewed through interviews conducted with patients and their caregivers. The data collected were analyzed using the Statistical Package for the Social Sciences (SPSS), version 26.0. A descriptive summary of the data was compiled through analysis, and the findings were reported in terms of frequencies and percentages. A multivariate logistic regression analysis was conducted to examine the association between the predictor variables and the outcome measure. Associations were evaluated using odds ratios (ORs) with their corresponding 95% confidence intervals (CIs).

**Results:**

Among patients with depressive disorders, the antidepressant adverse effects were observed in 82.9% of cases, with a 95% CI of 79.6–86.7. The most common adverse effects were weight gain (64%), nausea and vomiting (51%), dry mouth (49%), and headache (41%). Female was associated with a threefold increased risk of antidepressant drug adverse effects (AOR = 3.56, 95% CI: 2.73, 5.43). Additionally, being unemployed (AOR = 1.35, 95% CI: 1.30, 6.52), having a monthly income of 620 Ethiopian Birr (AOR = 4.53, 95% CI: 4.24, 9.58), limited physical activity (AOR = 4.52, 95% CI: 3.47, 11.87, and AOR = 3.03, 95% CI: 1.92, 4.32), consuming alcohol (AOR = 1.23, 95% CI: 1.20, 3.03), and using drugs for more than 2 years (AOR = 1.37, 95% CI: 1.25, 3.58) were significantly linked to antidepressant drug adverse effects in patients with depressive disorders.

**Conclusion:**

Depressive disorder treatment often encounters side effects from antidepressants. Women who are unemployed, have a monthly income of less than 620 Ethiopian Birr, engage in a limited level of physical activity, consume alcohol, and have used drugs for more than 2 years experienced significant adverse effects from antidepressants.

## 1. Introduction

Mental health challenges like depression pose a significant global health burden, affecting an estimated 4.4% world population as of 2015, with depression being a major factor in the total burden of mental illnesses [1]. Depression substantially impairs individuals and communities by compromising functional capabilities. In the United States, nearly 7.1% of adults have had at least one episode of depressive disorder, and 63.8% of them experienced severe impairment [2]. Globally, depression accounts for 7.5% of all years lived with disability, ranking as the leading cause of nonfatal health loss worldwide [3].

Antidepressant medications are standard treatment for depression disorder, alleviate symptoms of low mood and anxiety, and help prevent relapse [4, 5]. Besides depression, antidepressants are also indicated for insomnia, eating disorders, smoking cessation, pain, attention‐deficit/hyperactivity disorders, and migraines [6]. Common pharmacologic treatments include tricyclic antidepressants (TCAs), selective serotonin reuptake inhibitors (SSRIs) and serotonin‐norepinephrine reuptake inhibitors (SNRIs) [7]. Treatment usually begins with monotherapy, with combinations considered if initial therapy fails [8]. Duration varies depending on illness severity, from several months to years [9]. However, evidence suggests antidepressants provide only modest benefit over placebo and may be less effective or more harmful compared to psychotherapy or cognitive behavioral therapy, warranting careful risk–benefit assessment [10, 11].

In Ethiopia, older antidepressants such as TCAs remain widely used because of lower cost and greater availability. Essential medications, especially newer psychotropic drugs, often have limited availability and affordability in public and private pharmacies [12]. Economic constraints, supply chain limitations, and scarcity of mental health specialists outside urban areas further limit access to newer treatments, making TCAs a pragmatic choice in many settings. Sociodemographic factors such as female gender, marital status, occupation, income, and education level have been associated with higher depression risk and antidepressant use [13].

Adverse drug reactions from antidepressants can cause significant morbidity, hospitalizations, and treatment discontinuation, with relapse occurring in about one‐fourth of users during follow‐up [14, 15]. Studies report discontinuation rate ranging from 33% to 53%, with adverse effects between 23% tand 36% commonly cited as a reason [16]. Physicians often underestimate both the frequency and severity of these side effects, which critically affect treatment adherence and remission rates [17].

Common adverse effects leading to noncompliance include weight gain, sexual dysfunction, and fatigue [18]. Identifying and managing these adverse effects is essential for patient safety and optimal outcomes [19]. Given the limitations of prior single‐center studies with variable tools, this study is part of the project on the prevalence and determinants of antidepressant nonadherence among patients with major depressive disorder in Ethiopia: a multicenter cross‐sectional study and aims to assess the magnitude of antidepressant adverse effects and associated factors among depressive disorder patients, using a structured antidepressant side effect [20].

## 2. Methods

### 2.1. Study Setting

The research was carried out in the Amhara regional state, situated in the northwestern parts of Ethiopia, at the psychiatric clinics of Felege Hiwot, Debre Tabor, and Gondar Compressive and Specialized Hospitals (CSHs). They are situated ~492–727 km away from Addis Ababa, the capital city of Ethiopia. In addition, over 2.5, 6.5, and 9 million residents of the catchment area are catered to by the hospitals, respectively. The psychiatry clinic offers both inpatient care to patients who have been admitted and outpatient follow‐up services for individuals with bipolar disorder, depression, substance abuse issues, and schizophrenia. At the Felege Hiwot, Debre Tabor, and Gondar psychiatry clinics, the number of patients with depressive disorders who had been taking antidepressants for 1 month or more was 265, 224, and 326, respectively.

### 2.2. Study Design and Period

A multicenter cross‐sectional hospital study took place from June 12, 2024, to November 13, 2024.

### 2.3. Source and Study Population

At the psychiatric clinics of Felege Hiwot, Debre Tabor, and Gondar CSH, individuals with depressive disorders who were on follow‐up were selected as the initial population, and those with depressive disorders who had been taking antidepressants for 1 month or more during the study period were identified as the study population.

### 2.4. Inclusion and Exclusion Criteria

The research encompassed all adult outpatients attending follow‐up appointments whose medical records unambiguously showed a diagnosis of depression, who were tranquil and taking antidepressant medication for a period 1 month or more. Patient refusal, incomplete or unavailable medical records, patients or caregivers with a severe general medical condition and who were unable to converse were excluded from participation.

### 2.5. Study Variables

Patient‐reported adverse effects of antidepressant drugs were considered the dependent variables. The independent variables included sociodemographic and economic factors such as gender, age, income, occupation, area of residence, smoking status, khat use, alcohol consumption, and level of physical activity. Clinical factors were also included as independent variables, encompassing comorbidities, the underlying cause of depression, and the duration since initiation of antidepressant therapy.

### 2.6. Sample Size Determination and Sampling Technique

The sample size was calculated with the single population proportion formula in EPI Info version 7.2. With an assumed confidence level of 95% and a 5% margin of error, an estimated 69% of patients with depressive disorder at Addis Ababa, Ethiopia [21], experienced antidepressant adverse drug reactions, resulting in an effective sample size of 534 after accounting for 10% nonresponses.

n=Zα/22×p×1−pd2=1.962×0.5×10.69−0.052×210 dign effect+% n=534.

**Figure 1:**
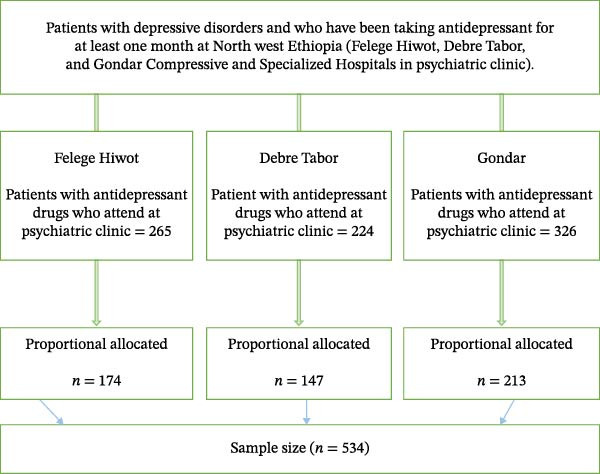
Schematic representation of sampling procedures of patients who received antidepressant drugs in psychiatric clinics in public hospitals, Ethiopia, 2024 (*N* = 534).

A straightforward random sampling method was employed. During the data collection period, patient records received at the clinic were examined to determine their eligibility. New patients were differentiated from the charts, and participants in the study were selected through a random process. When a patient is absent at the time the chart is received, an alternative patient who satisfies the necessary criteria is selected. The patients listed in subsequent records served as the reference point for the study’s sample selection (Figure 1).

### 2.7. Data Collection Procedure

Prospective data were collected from interviews with patients and their prescribers through communication with psychiatrists. The data was initially processed in English, subsequently translated into Amharic, and then retranslated back into English. Data were collected on sociodemographic and economic characteristics, as well as clinical‐related factors (age, gender, education, marital status, religion, occupation, and residence), and information regarding antidepressant prescription medications. The antidepressant side effect checklist (ASEC), developed by the Royal College of Psychiatrists, was utilized to categories reported side effects and encompasses a comprehensive list of side effects linked to antidepressant medications, as well as classifying a mental state examination (MSE) as mild, moderate, or severe [22]. A team of nine psychiatrists, consisting of three at each of three hospitals, gathered the data. The lead investigators and their coinvestigators offered supervision throughout the duration of the data collection process.

### 2.8. Data Quality Assurance

The study tools were developed based on a review of the existing English literature, specifically references, the Naranjo Probability scale [23], ASEC [24], and adverse drug reaction severity score [25]. The data were gathered in Amharic, taking into account the patients’ level of language proficiency. A pretest of the questionnaires was carried out on 10% of patients with depression who were being treated with antidepressants at the psychiatric clinic of Dessie Hospital prior to data collection, to prevent cross‐contamination of information and to verify the questionnaire’s linguistic clarity and consistency. Ambiguous terms in the questionnaire were clarified following the pretest. Prior to the patients’ departure from the psychiatric clinic, the questions were reviewed for thoroughness and revised accordingly. To maintain accuracy and consistency, the collected data was input into a computer using a double data entry approach.

### 2.9. Data Processing and Analysis

The data was cleaned for thoroughness, assigned codes, input into EpiData, then moved over, and examined with the Statistical Package for the Social Sciences (SPSS) version 26.00. The descriptive analysis was summarized using frequency and percentage. A bivariate analysis was employed to examine the association between the independent variable and the outcome variable. Variables with a *p*‐value lower than 0.25 in the bivariate analysis were selected for further multivariate analysis. The model’s significant variables were initially identified by filtering the variables through the backward method, followed by the application of the entry technique to the remaining variables. Model fitness was assessed using the Hosmer–Lemeshow test. After the correction, a variable independent of the others was deemed significantly associated with the dependent variable if it had a *p*‐value of 0.05 or less. The results were presented along with an adjusted odds ratio (OR) and a 95% confidence interval (CI).

### 2.10. Ethical Approval

The University of Gondar College of Medicine and Health Sciences’ School of Pharmacy’s Ethics Review Committee approved the ethics protocol with reference number SOP 088/2024 on May 22, 2024. Data collection began once consent had been obtained from each facility. All participants gave their informed consent to take part in the survey.

## 3. Operational Definition

Antidepressant medications were utilized to treat depression through a combination of pharmacological and nonpharmacological approaches. Pharmacologic treatments that are frequently recommended include TCAs, selective SSRIs, and SNRIs [8].

Patients who take antidepressants have reported a range of unwanted effects: These reported effects include any documented and anticipated medication side effects or adverse drug reactions that not only make it difficult for patients to stick to their treatment plans but may also lead to the relapse of their condition and reduce their overall quality of life. The physical and psychological side effects of antidepressant treatment significantly impact the therapeutic experience and outcomes for individuals receiving this type of treatment [26].

## 4. Results

### 4.1. Sociodemographic and Clinical Characteristics of Study Participants

Of the initial sample of 534 patients, 422 patients with depressive disorders attending psychiatry clinics in the three CSH hospitals were analyzed, yielding a response rate of 80%, while 112 (21.0%) were excluded due to nonresponse. Reasons for nonresponse included patient refusal, incomplete or unavailable medical records, and exclusion based on comorbidities or concomitant medications that met the exclusion criteria. More than half of the participants were female (279; 66.4%). The mean age of participants was 30.2 years (SD ± 11.9). The majority were unemployed (370; 88.1%), and approximately one‐quarter (120; 28.4%) reported a monthly income of less than 620 ETB. Regarding lifestyle, 143 participants (33.9%) reported moderate levels of physical activity, while 179 (42.4%) were alcohol users. In terms of treatment history, most patients (158; 37.6%) had a follow‐up duration of 1–2 years with antidepressant medications, as shown in (Table 1).

**Table 1:** Sociodemographic and clinical characteristics of patients in psychiatric clinics in public hospitals, Ethiopia, 2024 (*N* = 422).

Variables	Category	*n* (%)
Sex	Male	143 (33.6)
	Female	279 (66.4)
Age	20−37 years	144 (34.1)
	38−46 years	129 (30.6)
	47−54 years	73 (16.8)
	55–75 years	76 (18)
Marital status	Single	48 (11.4)
	Married	318 (75.4)
	Divorced	35 (8.3)
	Widowed	21 (5.0)
Education	Literate	234 (57.8)
	Illiterate	186 (44.2)
Employment	Employed	150 (43.4)
	Unemployed	370 (88.1)
Residency	Rural	188 (44.5)
	Urban	234 (55.5)
Economic income monthly (ET Birr)	<620 birr	120 (28.4)
	630−760 birr	96 (22.7)
	780−1220 birr	102 (24.2)
	1230−6000 birr	104 (24.6)
Smoking status	Smoker	193 (45.7)
	Nonsmoker	229 (54.3)
Khat (*Catha edulis* is a plant or tree with leaves as used as a stimulant drug)	Yes	76 (18.0)
	No	346 (82.0)
Alcohol	Yes	179 (42.4)
	No	243 (57.6)
Level of physical activity	None	31 (7.3)
	Minimal	116 (27.5)
	Moderate	143 (33.9)
	Active	132 (31.3)
Comorbidity diseases	Yes^a^	53 (12.6)
	No	369 (87.4)
Duration since starting the drugs (year)	<One	120 (28.6)
	One–two	158 (37.6)
	>Two	142 (33.8)
The reason for your depression	Family history	93 (22.0)
	Loneliness	142 (33.6)
	Stressful events	65 (15.4)
	Illness	55 (13.0)
	Alcohol and drugs	37 (8.8)
	Pregnancy	24 (5.7)

^a^Hypertension, diabetes, epilepsy ischemic heart disease, thyroid disorder.

### 4.2. Patient‐Reported Pattern of Antidepressant Drugs Among Study Participants

**Figure 2:**
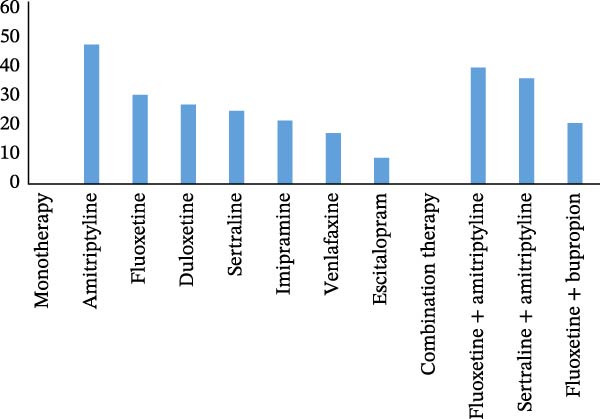
Patient‐reported antidepressant drugs (%) used during the follow‐up in psychiatric clinics in public hospitals, Ethiopia, 2024 (*N* = 422).

Amitriptyline, a TCA, was the most frequently prescribed medication as a single treatment option, accounting for 47.9% of cases, while fluoxetine and duloxetine, a selective SSRI and SNRI, respectively, came in second at 30.6% and 27.3%. Amitriptyline in combination with fluoxetine is commonly prescribed in 40% of cases, as shown in (Figure 2).

### 4.3. Patient‐Reported Antidepressant Drug Side Effects Among Participants

**Figure 3:**
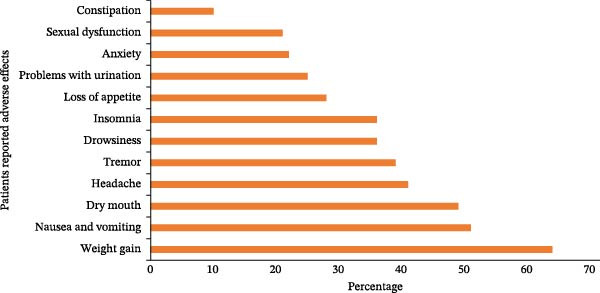
Patient‐reported antidepressant drug adverse effects in psychiatric clinic in public hospitals, Ethiopia, 2024 (*N* = 422).

The majority of participants experienced side effects from antidepressant medication, with the most frequent issues being weight gain (64%), nausea and vomiting (51%), dry mouth (49%), and headache (41%), as shown in (Figure 3).

### 4.4. The ADR Severity Score and the Naranjo Probability Scale of the Study Participants

A moderate adverse drug reaction prevalence (126; 30%) was observed, surpassing that of mild (123; 29.3%) and severe (112; 26.6%) cases. The Naranjo score indicated that about 218 (51.7%) of the reported ADRs were likely possible, while 112 (26.5%) were probably related to the drug (Table 2).

**Table 2:** Severity and probability of antidepressant adverse effects of patients received antidepressant medication at psychiatric clinic in public hospitals, Ethiopia, 2024 (*N* = 422).

Variable	Category	*n* (%)
	Absent	61 (14.1)
Adverse drug reaction severity	Mild	123 (29.3)
	Moderate	126 (30)
	Severe	112 (26.6)
Naranjo probability scale	Absent	92 (21.8)
	Possible	218 (51.7)
	Probable	112 (26.5.)

### 4.5. Prevalence of Patient‐Reported Antidepressant Drug Adverse Effects

**Figure 4:**
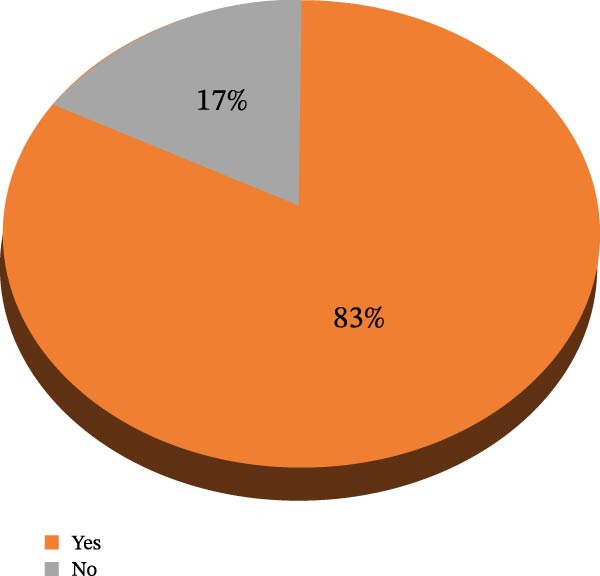
Prevalence of patient‐reported antidepressant drug adverse effects in psychiatric clinics in public hospitals, Ethiopia, 2024 (*N* = 422).

Three hundred and 50 participants in the current study reported experiencing antidepressant drug adverse effects, with a prevalence of 83%, as indicated in the 95% CI of 79.6% −86.7% (Figure 4).

### 4.6. Factors Associated With Patient‐Reported Antidepressant Drug Adverse Effects

Bivariate analysis revealed that sex, age, employment status, monthly income, level of physical activity, alcohol use, and years since starting drug use were considered candidate variables, subsequently entered into the multivariable logistic regression model. In the multivariate analysis, being female (AOR of 3.56, 95% CI: [2.73, 5.43]), being unemployed (AOR of 1.35, 95% CI: [1.30, 6.52]), having a monthly income of less than 620 ETBirr (adjusted OR of 4.53, 95% CI: [4.24, 9.58]), and engaging in little to no physical activity (AOR of 4.52, 95% CI: [3.47, 11.87], and 3.03, 95% CI: [1.92, 4.32]) were significantly associated with antidepressant drug adverse effects. Additionally, being an alcohol drinker (AOR of 1.23, 95% CI: [1.20, 3.03]) and experiencing more than 2 years of drug use since starting (AOR of 1.37, 95% CI: [1.25, 3.58]) were also found to be significantly associated with antidepressant drug adverse effects (Table 3).

**Table 3:** Factors associated with patient‐reported antidepressant drug adverse effects in psychiatric clinic in public hospitals, Ethiopia, 2024 (*N* = 422).

Variable	Category	Antidepressant drug side effects		COR (95% CI)	AOR (95% CI)
		Yes	No		
Sex	Male	84 (58.3)	59 (41.3)	1	1
	Female	238 (85.3)	41 (14.7)	4.08 (2.55–6.52)	3.56 (2.73−5.43) ^∗∗^
Age	20−37 years	105 (65.2)	56 (34.8)	1	1
	38−46 years	100 (82.6)	21 (17.4)	2.54 (1.43−4.45)	—
	47−54 years	54 (85.7)	9 (14.3)	3.20 (1.47−6.96)	—
	55−75 years	62 (81.6)	14 (18.4)	2.36 (1.22−4.59)	—
Employment status	Employed	34 (65.4)	18 (34.6)	1	1
	Unemployed	316 (85.4)	54 (14.6)	3.10 (1.63−5.88)	1.35 (1.30−6.52) ^∗∗^
Monthly income (ET Birr)	<620	111 (92.5)	9 (7.5)	6.81 (3.09−14.99)	4.53 (4.24−9.58) ^∗∗^
	630–760	84 (87.5)	12 (12.5)	3.87 (1.87−7.99)	—
	780–1220	88 (86.3)	14 (13.7)	3.74 (1.74−6.94)	—
	1230–6000	92 (88.4)	12 (11.6)	1	1
Level of physical activity	None	28 (90.3)	3 (9.7)	3.91 (1.12−13.63)	4.52 (3.47−11.87) ^∗∗^
	Minimal	103 (88.8)	13 (11.2)	3.32 (1.67−6.61)	3.03 (1.92−4.32) ^∗^
	Moderate	126 (88.1)	17 (11.9)	3.11 (1.66−5.83)	—
	Active	93 (70.5)	39 (29.5)	1	—
Alcohol drink	Drinker	139 (77.7)	40 (22.3)	1.81 (1.14−3.17)	1.23 (1.20−3.03) ^∗^
	Nondrinker	161 (66.3)	82 (33.7)	1	1
Smoker status	Smoker	147 (76.2)	46 (23.8)	2.443 (1.444−4.133)	—
	Nonsmoker	133 (58.1)	96 (41.9)	1	1
Duration since starting of drugs (year)	<1	88 (73)	33 (27)	1	1
	1–2	135 (85.4)	23 (14.6)	2.176 (1.199−3.949)	—
	>2	126 (88.7)	16 (11.3)	2.92 (1.515−5.626)	1.37 (1.25−3.58) ^∗^

*Note:* Hosmer and Lemeshow goodness of fit *p* = 0.741.

^∗^ *p* < 0.05.

^∗∗^ *p* < 0.01.

## 5. Discussion

Depression is a widespread psychiatric disorder that necessitates thorough and integrated medical treatment. The conventional approach typically begins with the concurrent use of antidepressant medication and psychotherapy, with the anticipation of symptom alleviation. However, the clinical course is closely linked to the profile of antidepressant side effects [27]. Our study seeks to examine the distinct patterns of adverse reactions that are linked to antidepressant medication use in patients suffering from depression, taking into account their significant impact on treatment outcomes.

Our aim in this investigation is to identify the factors that contribute to the manifestation of these side effects, providing valuable information for tailoring treatment approaches and improving patient care within the realm of depression management.

Research has revealed that the incidence of antidepressant medication side effects is a significant concern, with a striking (82.9%) prevalence rate, specifically a 95% CI of (79.6–86.7). Notably, this rate is significantly higher than those found in other studies, including one conducted in Nepal (74.13%) [24], Ethiopia (69%) [21], and Canada (38%) [28]. The observed discrepancy may be attributed to various factors, including differences in client history, study duration, and the instrument employed. The present investigation displayed a greater prevalence due to its emphasis on antidepressant medications comprising tricyclic, SSIRs, and SNIRs, which exhibited the most significant side effects and predominantly occurred in rural and low‐income regions. Previous studies have primarily focused on the side effects and adverse reactions associated with SSRI medications. This prevalence outcome is consistent with a previous study in Ethiopia, which reported an incidence of 85.7% [29]. The adverse effects reported were consistent across every site involved in the study. No variations in adverse effects could be attributed to factors unique to a particular site, such as differences in patient demographics, local prescribing practices, or healthcare delivery systems.

The majority of antidepressant prescriptions in this study were allocated as follows: amitriptyline (TCA) held the highest percentage at 47.9%, followed by fluoxetine (SSRI) at 30.6%, and duloxetine (SNRI) at 27.3%. This outcome inline the study conducted article review in Nepal, that (TCAs) constituted the majority of prescribed medications at 61.3%, with amitriptyline accounting 60.6%, fluoxetine (SSRI) 38.7% and sertraline (SNRI) at 20.2% [7]. Furthermore, a previous study in a comparable situation discovered that in Nigeria, the most prescribed monotherapy medicine was amitriptyline (28.1%), followed by fluoxetine (27.2%) [26]. However this finding contrary study done in Nepal, fluoxetine (SSRI; 58.04%, 50.27%) was followed by sertraline (SSRI; 13.79,40.29%), and amitriptyline (TCA; 9.77, 25.31%) [30]. Similarly In Malaysia, SSRIs (fluoxetine; 72.9%), followed by amitriptyline (TCAs; 10.3%), and Sertraline (SNRIs; 8.4%) [31]. The findings of our research indicate that TCAs are the most commonly prescribed antidepressants for treating depression, surpassing selective SSRIs and other SNRIs. The lack of affordability and availability might be the reason. In contrast to other studies, it was found that when treating depression, SSRIs are more frequently prescribed than TCAs, SNRIs, and other atypical antidepressants [7]. In less developed countries, affordability is a crucial factor in sustaining therapy because numerous low‐income households are unable to afford expensive medications [32]. The bulk of the population must rely on government‐backed insurance plans to access medications for their treatment [33]. Individuals often obtain their prescribed antidepressants from government‐run hospitals, as they are more economical compared to retail pharmacies. Regional disparities combined with cultural differences and varying national economies result in significant disparities in antidepressant prescribing practices.

In our study, patients experienced a total of 12 side effects associated with antidepressant drugs, with weight gain being reported by 15.2% of patients, followed by nausea and vomiting in 12.1%, dry mouth in 11.7%, and headache in 9.8% as the most frequently occurring concerns. The study in India yielded a comparable result, where the most frequent symptom reported was dry mouth, with nausea and tremor being the next most common complaints, specifically noted in 47 participants [34]. In Nepal, dry mouth, weight gain, drowsiness, and blurred vision are commonly reported side effects, along with problems related to sexual function [35]. In comparison to other studies, our results differ from those in Nepal, where insomnia and anxiety were the most frequently occurring side effects (17.05%), while dry mouth and weight gain occurred at lower rates of 10.85% and 10.07%, respectively [36]. Variations in reported side effects across different cultures highlight the need to take into account regional and cultural elements that can affect the tolerance and expression of side effects associated with antidepressant medication. Our study offers valuable insights into the lived experiences of individuals undergoing antidepressant therapy based on the patterns of reported side effects. These insights provide a more detailed understanding of how antidepressant therapy affects patients’ quality of life and help inform targeted strategies to reduce side effects in various groups of people [37]. In order for these side effects to be manageable, special attention needs to be given to facilitate collaborative decision‐making and boost the likelihood of an individual receiving effective medication with minimal adverse effects [38].

All side effects should be evaluated with equal importance, as many were classified as moderate to likely on both the ASEC and Naranjo scales. Most reported side effects were not associated with antidepressant treatment being stopped. Some adverse effects can be significant predictors of individuals stopping use of antidepressants. When selecting antidepressants, healthcare professionals need to take into account various factors, including adverse effect profiles, costs, safety profiles, patients’ medical histories of previous pharmaceutical treatments, and personal preferences [39]. Furthermore, antidepressant drug side effects are the key predictors of antidepressant‐related nonadherence, which is likely to be particularly high among depressed people [40].

This research investigation revealed a number of variables linked to the side effects of antidepressants. Females who are unemployed, earn less than 620 ETBirr/month, do not engage in regular exercise, consume alcohol, and have been taking medication for more than 2 years are significantly more likely to experience adverse effects. Among females, the risk of experiencing antidepressant side effects was 3.56 times greater than among males. Female subjects also experienced side effects [41]. The gender‐based connection highlights the need to take into account sex‐specific factors when anticipating and managing side effects linked to antidepressant treatment. A thorough comprehension of these associated factors is crucial in designing interventions that can counteract adverse effects and enhance overall treatment outcomes for a wide range of patient groups [42]. In this study, individuals who were unemployed were found to have a higher risk of experiencing adverse antidepressant effects compared to those who were employed. Monthly income less than 620 ETBirr had 4.53‐time higher odds than income more than 630 ETBirr. Studies have found that individuals with lower socioeconomic status are more likely to discontinue antidepressant therapy prematurely, with earlier termination rates noted at [43, 44]. Furthermore, research indicates that individuals of lower income are more frequently prescribed TCAs than those with higher incomes, which can lead to increased adverse effects [45].

Individuals with little to no physical activity had a 4.52‐ and 3.03‐time greater risk of experiencing adverse effects from antidepressants compared to those with a moderate to active level of physical activity among patients with a depressive disorder. Research confirming the comparison of exercise and antidepressant treatments for depression indicates that exercise can be a useful component in the treatment of depression, potentially reducing the negative effects associated with antidepressant medications [46]. Evaluating the advantages of combining physical activity with antidepressants in treating depression, this synergy’s benefits on both a neurobiological and clinical level are examined, focusing primarily on the effects of physical exercise on affective and cognitive symptoms in depression [47]. Individuals who consume alcohol have a 1.23‐fold increased risk of experiencing antidepressant‐related side effects compared to those who do not drink. Previous research has demonstrated that alcohol depresses the human neurological system, causing a decrease in blood pressure, breathing rate, and heart rate. Additionally, it can worsen depressive symptoms and increase the likelihood of antidepressant side effects [48]. In this study, participants with a duration of more than 2 years since starting antidepressant medication had a 1.37 times greater risk of experiencing adverse effects compared to those with a duration of less than 2 years. Furthermore, long‐term antidepressant medication use has increased, and there is evidence of associated adverse effects [49].

This research possesses both positive and negative aspects. The current study, the first to comprehensively examine the factors of antidepressant adverse effects, can inform treatment decisions for both practitioners and patients. It is crucial for clinicians to fully inform patients about the broad range of potential adverse effects associated with antidepressants, including emotional blunting, suicidality, and withdrawal symptoms, which are frequently under‐communicated in practice. Empowering patients with this information supports shared decision‐making and may improve adherence and clinical outcomes. A major limitation of this study is that antidepressant withdrawal effects were not investigated. Withdrawal symptoms can be common and severe, impacting patient safety and treatment adherence. Patient self‐assessments of antidepressant side effects rely heavily on the honesty and trustworthiness of the individuals providing the information, which can potentially result in exaggerated or inaccurate scores. The study took place over a relatively brief timeframe, so any modifications to the treatment approach during that period remain unclear. Future research should include assessment of withdrawal phenomena using validated tools such as the Discriminatory Antidepressant Withdrawal Symptoms Scale (DAWSS) to comprehensively understand the adverse impact of antidepressants beyond initial side effects.

## 6. Conclusion

In Northwest Ethiopia, a significant number of patients with depressive disorders experience adverse effects from antidepressant use, with a staggering 80% of them being affected. A number of factors were significantly linked to these negative outcomes, such as being a female, being unemployed, having a low‐income, a lack or minimal level of physical activity, alcohol consumption, and prolonged use of antidepressants for over 2 years. The research highlights the necessity of targeted interventions to counterbalance undesirable consequences, particularly for those susceptible groups mentioned in the study. Monitoring patients’ progress regularly, educating them, and implementing supportive measures to manage lifestyle factors may lead to better patient outcomes and alleviate the negative impact of antidepressant side effects.

**Nomenclature**
AD: antidepressant medicines
AOR: adjusted odd ratio
ASEC: antidepressant side effect checklist
CSH: Compressive and sSpecialized Hospital
ETBirr: Ethiopian Birr
MSE: mental state examination
SNRIs: serotonin‐norepinephrine reuptake inhibitor
SSRIs: selective serotonin reuptake inhibitors
TCA: tricyclic antidepressant.

## Author Contributions

**Gashaw Sisay Chanie**: contribute in literature review, visualization, methodology, supervision, formal analysis, writing – original draft and manuscript writing – review and editing. **Gebremariam Wulie Geremew**: data collection, data curation, software, methodology. **Alemante Tafese Beyna**: contribute in literature review, data curation, supervision, formal analysis. **Melshew Fenta Misker**: contribute in literature review, data collection, methodology. **Habtamu Semagne Ayele**: data collection, investigation, data curation, methodology. **Abebe Worku Teshager**: proposal writing and data collection, data collection, investigation and writing – original draft. **Girma Medfu Takele**: proposal writing and data collection, data collection, methodology and writing – original draft. **Gizachew Kassahun Bizuneh**: contribute in literature review, methodology, supervision, formal analysis, writing – original draft. **Jember Azanaw**: contribute in literature review, methodology, supervision, formal analysis, writing – original draft and manuscript writing – review and editing. **Eyayaw Ashete Belachew**: proposal writing and data collection, formal analysis, and writing – original draft, and manuscript writing – review and editing. **Wudneh Simegn**: contribute in literature review, methodology, supervision, formal analysis, and writing – original draft, and manuscript writing – review and editing.

## Funding

No funding was received for this research.

## Ethics Statement

The University of Gondar College of Medicine and Health Sciences School of Pharmacy’s Ethical Review Committee, through SOP (088/2024) dated May 22, 2024, provided ethics approval. Following consent from each facility, the data were gathered. All participants gave their informed consent prior to taking part in the survey.

## Consent

All participants gave their informed consent prior to taking part in the survey.

## Conflicts of Interest

The authors declare no conflicts of interest.

## Data Availability

The data that support the findings of this study are available in the Supporting Information of this article.
